# Exploring how falls prevention practitioners assess and manage concerns about falling

**DOI:** 10.1007/s41999-024-01127-2

**Published:** 2024-12-17

**Authors:** Bianca Nicklen, Kim Delbaere, Toby J. Ellmers

**Affiliations:** 1 Department of Brain Sciences, Centre for Vestibular Neurology, Charing Cross Hospital, Imperial College, London, W6 8RP UK; 2 Falls, Balance and Injury Research Centre, Neuroscience Research Australia, Randwick, NSW Australia; 3 Australia and School of Population Health, University of New South Wales, Kensington, NSW Australia

**Keywords:** Concerns about falling, Older adults, Falls prevention

## Abstract

**Background:**

Concerns about falling (CaF) are common in older adults. They can lead to various negative outcomes, including an increased risk for future falls. The Worlds Falls Guidelines recently published recommendations for assessing and treating CaF. However, the extent to which these guidelines have been adopted into falls prevention practice (in addition to the barriers preventing implementation) are currently unknown.

**Methods:**

A cross-sectional survey was completed by 114 healthcare professionals working in falls prevention and rehabilitation services in the UK and Ireland. The survey explored their experiences and perceptions regarding the management and assessment of CaF.

**Results:**

Only 39% of respondents reported using the Falls Efficacy Scale-International (FES-I), a tool recommended by the World Falls Guidelines for assessing CaF. Healthcare professionals in hospital settings were significantly less likely to use the FES-I compared to those working in the community (*X*^2^ = 6.324, *p* = 0.043). While there was no significant difference between settings regarding the type of intervention used to manage CaF, only about 50% of participants adopted a holistic approach combining physical and psychological strategies as recommended by the World Falls Guidelines. The most commonly identified barriers to clinical management of CaF were a lack of both time and perceived effective interventions, particularly for those working within hospital settings (*X*^2^ = 6.209, *p* = 0.013 and *X*^2^ = 3.752, *p* = 0.053, respectively).

**Conclusion:**

These findings indicate low levels of adoption of the World Falls Guidelines recommendations for assessing and managing CaF, especially in hospital settings. Future work should focus on addressing these barriers to maximise the clinical adoption of these recommendations.

**Supplementary Information:**

The online version contains supplementary material available at 10.1007/s41999-024-01127-2.

## Key summary points

**Aim:**

The 2022 World Falls Guidelines presented recommendations on the clinical assessment and management of concerns about falling. The uptake and barriers surrounding these recommendations are currently unknown.

**Findings:**

These findings highlight low uptake of these clinical guidelines within falls prevention services in the UK and Ireland—particularly for those working in hospital settings.

**Message:**

Efforts should be placed on addressing the barriers to implementing these clinical guidelines (especially in hospital settings) to maximise their uptake.

**Supplementary Information:**

The online version contains supplementary material available at 10.1007/s41999-024-01127-2.

## Introduction

Approximately 30% of community-dwelling older adults experience a fall each year, leading to high levels of hospitalisation, morbidity and mortality [1, 2]. As such, many older adults develop concerns about falling (CaF) – defined as “lasting dread and apprehension about situations that are believed to threaten or challenge balance” [3]. CaF can result in an acute emotional response (i.e. fear or anxiety about falling) when older adults perceive that their balance is threatened [3, 4]. CaF can lead to further negative outcomes including activity restriction, social isolation, poorer rehabilitation outcomes and increased fall risk [5–9]. These potential outcomes underscore the need for effective CaF assessment and management by healthcare professionals.

The recently published World Falls Guidelines (WFG) provide specific guidance on the clinical assessment and management of CaF [10]. WFG recommended using either the full or short version of the Falls Efficacy Scale–International (FES-I) scale to assess CaF due to its high reliability and validity [11]. By assessing CaF, healthcare professionals can better understand their patients’ perceptions of fall risk, which provides important insight into their openness and willingness to participate in different interventions [10]. Once identified, CaF can be addressed through physical intervention (e.g., balance training or exercise) [12], psychological strategies (e.g., cognitive behavioural therapy (CBT)) [13], or a combination of both [14, 15]. However, targeting CaF through either physical or psychological strategies alone has shown inconsistent results, with any positive effects being small-to-moderate in size and short-lived [12–14, 16]. Recent systematic reviews have instead highlighted the efficacy of holistic interventions that combine both physical and psychological strategies [14, 15, 17], which the WFG also endorses for CaF management [5, 10].

Despite the publication of the WFG, there is limited evidence on how healthcare practitioners currently assess and manage CaF, nor the extent to which the WFG recommendations on this topic have been adopted into falls prevention practice. Further, whilst research has explored barriers faced when implementing falls prevention strategies more broadly (e.g. [18, 19]), barriers specific to the clinical management of CaF are underexplored. Understanding these challenges is crucial for both increasing the uptake of the existing recommendations and ensuring that any future guidelines on this topic are designed in a way that maximises their implementation. Finally, there is also little understanding about how assessment and management approaches vary between different settings (e.g., community vs. hospital). For instance, although the WFG recommended the FES-I for hospital settings (based on initial evidence of its reliability and validity within these settings [11]), this scale was initially designed and validated for community use. Practitioners in hospital settings may therefore use alternative assessments such as single-item questions (e.g., “Are you worried about falling?” from the STEADI checklist [20]) or skip assessment altogether due to competing priorities [21]. Exploring management approaches across different contexts is particularly important as older adults with complex needs, who are more likely to have CaF [7, 22–24], often receive falls prevention services within hospital settings [25, 26].

This study therefore aims to: (i) investigate how CaF is currently being assessed and managed by falls prevention healthcare professionals; (ii) identify the key barriers these healthcare professionals face in assessing and managing CaF; and (iii) investigate any differences in assessment and management practices between hospital and community settings.

## Method

### Study design

The study used a cross-sectional survey design, targeting healthcare professionals working in fall prevention and/or management services in both hospital and community settings. To minimise heterogeneity from varying international healthcare systems, participation was limited to professionals currently working in the UK and Ireland. Participation involved completing an anonymous survey on a secure online platform (Qualtrics). No incentives were offered for completing the survey.

### Participants and sampling

Healthcare professionals currently working in fall prevention and management services were recruited via emails sent through falls prevention/ageing networks and social media posts describing the aims and purpose of the study (see Supplementary Material for the recruitment materials). With 20 main survey items, a target sample size of N = 100 was based on the recommended sample-to-item ratio for survey completion of 5-to-1 [27]. A total of 114 participants completed the survey (*M*_age_ = 40.2 years, *SD* = 10.7); female, N = 97 (85%)). Further demographic information is provided in Table 1.

**Table 1:** Table showing the demographic and background information  of the participants

	Total N = 114 (%)	Hospital setting N = 49 (%)	Community setting N = 52 (%)	Combined role N = 13 (%)
Age (N = 100)				
≤ 30	8 (8.0)	5 (12.5)	2 (4.2)	1 (8.3)
31–40	26 (26.0)	14 (35.0)	9 (18.8)	3 (25.0)
41–50	35 (35.0)	12 (30.0)	19 (39.6)	4 (33.3)
51–60	19 (19.0)	8 (20.0)	9 (18.8)	2 (16.7)
> 60	12 (12.0)	1 (2.5)	9 (18.8)	2 (16.7)
Gender (N = 114)				
Female	97 (85.1)	39 (79.6)	48 (92.3)	10 (76.9)
Male	17 (14.9)	10 (20.4)	4 (7.7)	3 (23.1)
Highest qualification obtained (N = 113)				
NVQ	7 (6.2)	3 (6.1)	4 (7.8)	0 (0.0)
Undergraduate degree	44 (38.9)	14 (28.6)	24 (47.0)	6 (46.2)
Masters (conversion)	2 (1.8)	2 (4.1)	0 (0.0)	0 (0.0)
PhD	5 (4.4)	3 (6.1)	2 (3.9)	0 (0.0)
Medical doctor	6 (5.3)	4 (8.2)	0 (0.0)	2 (15.4)
Other	16 (14.2)	7 (14.3)	8 (15.7)	1 (7.7)
Masters (other)	33 (29.2)	16 (32.7)	13 (25.5)	4 (30.8)
Number of years in current role (N = 113)				
< 5	43 (38.1)	26 (53.1)	13 (25.5)	4 (30.8)
5–10	33 (29.2)	8 (16.3)	20 (39.2)	5 (38.5)
11–15	16 (14.2)	7 (14.3)	8 (15.7)	1 (7.7)
16–20	7 (6.2)	2 (4.1)	4 (7.8)	1 (7.7)
≥ 20	14 (12.4)	6 (12.3)	6 (11.8)	2 (15.4)
Professional role (N = 113)				
Occupational therapist	9 (8.0)	5 (10.2)	4 (7.8)	0 (0.0)
Occupational therapist assistant	1 (0.9)	1 (2.0)	0 (0.0)	0 (0.0)
Physiotherapist	61 (54.0)	24 (49.0)	28 (54.9)	9 (69.2)
Physiotherapist assistant	3 (2.7)	1 (2.0)	2 (3.9)	0 (0.0)
Geriatrician	9 (8.0)	7 (14.3)	0 (0.0)	2 (15.4)
Psychologist	1 (0.9)	1 (2.0)	0 (0.0)	0 (0.0)
Nurse	6 (5.3)	3 (6.1)	2 (3.9)	1 (7.7)
Exercise/postural therapist	11 (9.7)	0 (0.0)	11 (21.6)	0 (0.0)
Other	12 (10.6)	7 (14.3)	4 (7.8)	1 (7.7)
Frequency they see older adults in their role (N = 105)				
Daily	74 (70.5)	38 (80.9)	29 (61.7)	7 (63.6)
Multiple times p/w	26 (24.8)	8 (17.0)	15 (31.9)	3 (27.3)
Once a week	2 (1.9)	0 (0.0)	1 (2.1)	1 (9.1)
Once a month	3 (2.9)	1 (2.1)	2 (4.3)	0 (0.0)

All information listed is split into hospital, community, and a combination of both settings

### Ethics

Institutional ethical approval for the study was obtained from the Imperial College Research Ethics Committee (#6448485) and all participants provided informed digital consent prior to participation.

### Role of the funding source

The funders played no role in the design, conduct, or reporting of this study.

### Survey design and validity

The survey items were developed through discussions with healthcare professionals and experts working in falls prevention (all of whom had experience of working in both hospital and community settings). The Checklist for Reporting Results of Internet E-Surveys (CHERRIES) was followed to ensure survey quality (see Supplementary Material). To maximise completion rates, the Dillman Method was used [28, 29]. This included designing the survey to be respondent-friendly, keeping the completion time to 15–20 min and sending follow-up reminder emails one month after initial distribution. Cognitive Interviewing was also conducted to ensure the validity of the survey [30, 31]. This qualitative method helps understand how target population interpret and respond to surveys, ensuring that individual survey questions fulfil their intended purpose [30, 32]. Two experienced healthcare professionals (10+ years’ experience in falls prevention and rehabilitation) participated in the Cognitive Interviewing. Using the ‘thinking-aloud’ paradigm [30, 31], the healthcare professional completed the survey whilst elaborating on their interpretation and responses to the questions, providing explanations for their answers selected, and reporting any difficulties [32]. Feedback gained from the first interview was used to revise the survey before the second cognitive interview (e.g., problematic questions reworded based on feedback), and a second interview confirmed that the revisions were understood as intended. This approach minimises researcher bias (as the researcher takes a ‘hands off’ approach to directing discussions) and ensures that the survey accurately captured relevant insights. Cognitive Interviewing was stopped after the second interview, as feedback indicated that further new insights would be unlikely to emerge.

### Survey instrument

The survey consisted of 20 closed and open-ended questions, with each main question having several parts. Initial questions covered demographic information, including participants’ professional roles, settings (hospital versus community) and years of service. Specific questions focused on methods used to assess CaF (e.g., validated tools such as the FES-I versus informal methods), methods to manage CaF (i.e., physical, psychological, combined approaches or nothing), and perceived barriers to assessment and management (e.g., time constraints, communication issues, etc.). Participants were able to review and change their answers throughout the survey by using a back button. CaF assessment methods were considered ‘formal’ if they consisted of either validated questionnaires or single-item questions that could be scored (which have been shown to have moderate agreement with the FES-I [33]). In contrast, informal methods were considered any other method used to assess CaF, such as conversations with patients and/or caregivers. Open-ended questions allowed participants to elaborate on their answers or provide additional opinions. A full list of all questions and possible answers for closed questions can be found in the Supplementary Material.

### Data analysis

Descriptive statistics, frequencies and percentages were calculated to summarise variables, categorised by professional settings (hospital vs. community vs. combined role). Chi-Square tests compared key survey outcomes (modes of assessment, modes of intervention, barriers) between hospital (N = 49) and community settings (N = 52), excluding combined roles due to low participant numbers (N = 13). Significant Chi-Square tests were followed up with Bonferroni-corrected post-hoc tests (*Z*-test). All data was analysed using IBM SPSS 29.0.

## Results

### Demographic information

Data collection occurred between May 2023 and September 2023. Most respondents were physiotherapists (56.6%), falls prevention exercise therapists (9.7%) or occupational therapists (8.8%), with most having been in their role for five years or more (62.8%). Please see Table 1 for full breakdown of professional roles. Interestingly, a larger proportion of those working in hospital settings had been in their current role for less than 5 years compared to those in community settings (53.1% versus 25.5%; *X*^2^ = 6.72, *p* = 0.010)). The respondents were nearly evenly split between hospital (42.9%) and community settings (45.6%), with a smaller proportion working in both settings (11.4%).

### Clinical practice and responsibility in assessing and managing CaF

Most respondents (97.1%) reported seeing older adults at least once per week. The majority of participants (78.1%) reported that most older adults with CaF also had additional complex needs. The most common were frailty (27.1%), followed by cognitive impairment (19.3%) and dizziness (15.6%). When asked whose role it was to assess and manage CaF, no single profession was identified as being primarily responsible for assessing and administering CaF: GPs and geriatricians were selected most frequently at 26.3%, psychologists/psychiatrists as well as physiotherapists were reported similarly at 19.5% and 19.2%, respectively. This was followed by occupational therapists at 17.5% and finally by nurses at 13.7%. Additional comments revealed that CaF assessment should be completed by “all who come into contact” with the patient, emphasising that “falls is everyone’s business”.

### Assessing CaF

Most (87.5%) participants reported receiving formal training for assessing CaF, mainly through Continuing Professional Development (CPD; 25.6%), in-service training (24.4%) or webinars (19.2%). Most participants (97.0%) used these tools at the initial assessment. The frequency of assessment varied and depended on the type of care provided and the priority of CaF for continued assessment.

**Fig. 1:**
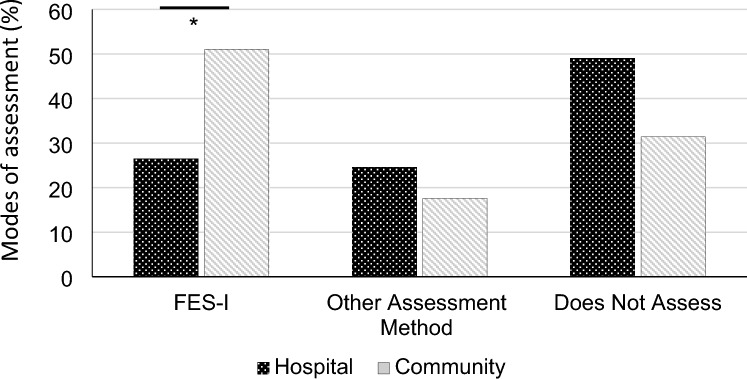
A comparison of the different methods used to assess CaF in older adults in hospital vs. community settings. FES-I includes both the full- and short-version of the tool. ‘Other assessment method’ includes both formal, validated tools (other than the FES-I) and informal methods (e.g., discussions with the patient). *p < 0.05 (Bonferroni-corrected)

Most participants (69.1%) reported using formal methods to assess CaF, with 53.3% using the WFG-recommended FES-I (short or full versions). There was a significant difference in the use of assessment tools between settings, with those in hospital setting (26.5%) less likely to use FES-I compared to those in community settings (51.0%) (*X*^2^ = 6.324, *p* = 0 0.043; Fig. 1). Key barriers to using formal CaF assessments included time constraints (26.8%), in addition to a lack of knowledge/training about the formal assessment tools available (26.8%). Other frequently used assessment tools included the Activity Balance Scale (9.1%), the Modified Falls Efficacy Scale (6.4%), and single-item questions assessing related constructs (e.g., fear or worries about falling; 7.3%). Some respondents preferred informal techniques, such as discussions with patients about their “fear of falling and the effects on their activities” or “confidence/anxiety regarding falls”.

### Managing CaF

Respondents felt somewhat (22.9%), fairly (55.4%), or completely confident (18.1%) in addressing CaF, with no significant differences between settings (*X*^2^ = 2.277, *p* = 0.320). Of those implementing interventions to manage CaF (81.6%), most used physical interventions targeting physical fall risk factors, either alone (38.7%) or combined with psychological methods (53.8%). A minority (5.4%) used psychological methods exclusively. Informal psychological methods included discussions and goal setting, while formal psychological methods (14.4%) often referred to psychological services. No significant difference was found between the types of interventions used in hospital and community settings (*X*^2^ = 2.255, *p* = 0.133). Examples of specific interventions used (provided via open-ended questions) can be found in the Supplementary Material.

**Fig. 2:**
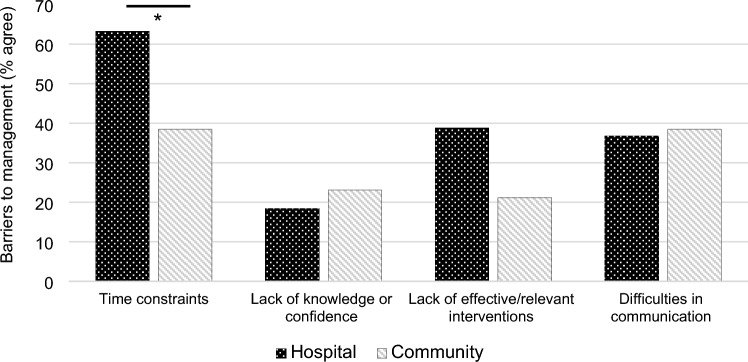
Barriers reported when addressing CaF between hospital vs. community settings; ‘Difficulties in communication’ relates to difficulties in communication both between departments, and between healthcare professionals and the patients themselves. Participants could select all that applied. *p < 0.05 (Bonferroni-corrected)

No significant difference was found between the type of intervention and its perceived effectiveness across settings (*X*^2^ = 2.325, *p* = 0.313). Most healthcare professionals believed current interventions were ‘somewhat’, ‘mostly’ or ‘very’ effective in addressing CaF (90.6%). The key barriers to managing CaF was time constraints, especially in hospital compared to community settings (63.3% vs 38.5%; *X*^2^ = 6.209, p = 0.013; Fig. 2). Other barriers included lack of perceived effective interventions, though this was not statistically significant between settings (*X*^2^ = 3.752, *p* = 0.053).

Open-ended comments highlighted further barriers such as ineffective referral processes, staffing pressures and/or lack of funding. One respondent noted: “We used to have psychology input in the centre, but this was only for a pilot then removed due to staffing pressures.” Where resources were available for addressing CaF, they were highly valued: “They appear to be very effective in reducing frequency and severity of falls as well as increasing confidence and social mobility.” Further comments highlighted lack of awareness about CaF and the “historical separation of mental and physical health concerns in treatment services” limiting access to psychological services.

## Discussion

This study explored how healthcare professionals currently assess and manage CaF among older adults. A main finding was that although two-thirds of the respondents used a formal method to assess CaF, only half used the WFG-recommended ‘gold-standard’ tools (short or full FES-I). Practitioners in hospital settings were significantly less likely to use these tools compared to those in the community—with time constraints identified as a key barrier within the hospital setting. The WFG recommended the FES-I due to its excellent reliability and validity [11]. Its widespread use could standardise outcome measures across different contexts—ensuring that CaF is assessed in a reliable and valid manner that is easily interpretable. However, although the short FES-I takes < 5 min to complete, the present findings suggest that even this brief assessment may be challenging in a busy hospital environment. Additionally, as the FES-I was designed for community settings, it may not capture hospital-specific activities leading to high number of missing responses in these settings (see [34]). Future work could therefore look to develop a shortened, hospital-specific assessment of CaF. Research has shown that FES-I scores collected in the hospital can independently predict rehabilitation outcomes at discharge and at 3-month follow-ups across geriatric disorders [9], underscoring the importance of assessing CaF in hospitals to support post-discharge planning.

The healthcare practitioners in this study demonstrated a high level of awareness and knowledge about CaF management: 87.5% reported having received formal training, 69.0% used formal measurement tools, and nearly all administered some form of intervention. Despite this high level of awareness surround CaF, there was generally low uptake of implementing WFG recommendations. The WFG recommended a multidisciplinary approach to CaF interventions, involving physical and psychological methods [10]. Research supports the effectiveness of multicomponent interventions for addressing CaF [14, 15, 35]. In the present study, just over 50% of respondents reported using holistic interventions as recommended by the WFG, with hospital practitioners slightly more likely to implement these approaches. This might be due to better access to psychological services in hospitals compared to community settings. Physical interventions focused mainly on strength and/or balance training, while informal psychological interventions often consisted of unstructured “conversations with patients”.

Time constraints and funding issues were identified as key barriers to effective CaF management, particularly in hospitals. Open-ended answers revealed that delivering structured psychologically interventions were especially challenging due to time pressures. Existing psychological strategies may not be suitable for current clinical practice as they often require multiple 60-min sessions [13, 36]. To address these barriers, new time-efficient strategies for holistic CaF management may need to be developed, such as training physiotherapists and occupational therapists to deliver psychological techniques alongside usual care. This approach—known as “psychologically informed practices”—is gaining acceptance in chronic pain management [37] and could be beneficial for CaF as well. Consideration should also be given to incorporating the complex needs of older adults when implementing interventions. Most respondents reported that most older adults with CaF had additional complex needs, such as frailty. This aligns with a recent systematic review that highlighted frailty as a risk factor for CaF [24]. A targeted CaF intervention for frail older adults could prevent a negative downward spiral of increasing CaF and frailty [38].

One limitation of this study is the potential sampling bias within the survey, as participants may already have an interest in, and knowledge about, CaF. Further, the recruitment methods (emails sent out through falls prevention/ageing networks and social media posts) prevented us from determining the response rate. The study was also restricted to falls prevention services within the UK and Ireland, making it difficult to generalise the findings to other countries. For instance, the American Geriatrics Society recommended that clinicians working in the USA assess CaF via the single-item question from the STEADI checklist (“Are you worried about falling?”) [39] rather than the WFG recommended FES-I. Although the FES-I has been translated into 43 languages, future work should nonetheless explore its usage across different international settings. Finally, as our sample size estimation was calculated based on the whole sample (rather than comparisons between community vs. hospital settings), some of our analyses may have been underpowered to detect subtle differences between groups.

Based on the findings from this survey, and in-line with the WFG, we recommend that healthcare professionals working in a hospital setting use the WFG-recommended short FES-I to assess CaF. Additionally, both hospital and community settings should explore ways to incorporate psychological strategies alongside their current physical-only interventions to bridge the gap between “physical” and “psychological” aspects of CaF management. However, future work is needed to identify the most effective way to integrate current psychologically informed interventions for CaF in hospital settings, as these interventions have thus far been primarily studied in community and care-home settings [40, 41]. Given that time constraints are a key barrier to the clinical management of CaF, focus should be placed on the development of new, time-efficient strategies that address CaF holistically (e.g., without requiring lengthy programs like an 8-week CBT course). Due to limited access to psychological professionals in falls prevention services, emphasis could instead be placed on training physiotherapists and occupational therapists to deliver psychological techniques alongside usual care (i.e., “psychologically informed practice” [37]).

## Conclusion

This study provides novel insights into healthcare professionals’ experiences and perceptions regarding the assessment and management of CaF. Key findings revealed that those working in hospital settings were significantly less likely to use the ‘gold standard’ assessment tools recommended by the WFG compared to those working in community settings. Although there were no significant differences between community and hospital settings in the type of interventions used, only half of participants adopted a holistic approach combining physical and psychological strategies. Key barriers to effective CaF management included time constraints, particularly in hospitals, and associated staffing pressures. Future work should focus on addressing barriers to maximise the clinical adoption of the WFG recommendations.

## Supplementary Information

Below is the link to the electronic supplementary material.

Supplementary file1 (DOCX 49 KB)

## Data Availability

All open ended responses are provided within the Supplementary Material. All other data can be obtained from the corresponding authors following reasonable request.

## References

[1] Salari N, Darvishi N, Ahmadipanah M, Shohaimi S, Mohammadi M (2022) Global prevalence of falls in the older adults: a comprehensive systematic review and meta-analysis. J Orthop Surg 17:334. 10.1186/s13018-022-03222-1 10.1186/s13018-022-03222-1 PMC9238111 35765037

[2] James SL, Lucchesi LR, Bisignano C, Castle CD, Dingels ZV, Fox JT et al (2020) The global burden of falls: global, regional and national estimates of morbidity and mortality from the Global Burden of Disease Study 2017. Inj Prev 26:i3. 10.1136/injuryprev-2019-043286 31941758 10.1136/injuryprev-2019-043286 PMC7571347

[3] Ellmers TJ, Wilson MR, Kal EC, Young WR (2023) The perceived control model of falling: developing a unified framework to understand and assess maladaptive fear of falling. Age Ageing 52:afad093. 10.1093/ageing/afad093 37466642 10.1093/ageing/afad093 PMC10355179

[4] Tashiro H, Hirosaki S, Sato Y, Ihira H, Toki M, Kozuka N (2025) Concern about falling is related to threat-induced changes in emotions and postural control in older adults. Gait Posture 115:1–6. 10.1016/j.gaitpost.2024.10.020 39454444 10.1016/j.gaitpost.2024.10.020

[5] Ellmers TJ, Freiberger E, Hauer K, Hogan DB, McGarrigle L, Lim ML et al (2023) Why should clinical practitioners ask about their patients’ concerns about falling? Age Ageing 52:afad057. 10.1093/ageing/afad057 37097766 10.1093/ageing/afad057

[6] Liu M, Hou T, Li Y, Sun X, Szanton SL, Clemson L et al (2021) Fear of falling is as important as multiple previous falls in terms of limiting daily activities: a longitudinal study. BMC Geriatr 21:350. 10.1186/s12877-021-02305-8 34098904 10.1186/s12877-021-02305-8 PMC8185919

[7] Scheffer AC, Schuurmans MJ, van Dijk N, van der Hooft T, de Rooij SE (2008) Fear of falling: measurement strategy, prevalence, risk factors and consequences among older persons. Age Ageing 37:19–24. 10.1093/ageing/afm169 18194967 10.1093/ageing/afm169

[8] Cumming RG, Salkeld G, Thomas M, Szonyi G (2000) Prospective study of the impact of fear of falling on activities of daily living, SF-36 scores, and nursing home admission. J Gerontol Ser A 55:M299-305. 10.1093/gerona/55.5.M299 10.1093/gerona/55.5.m299 10819321

[9] Denkinger MD, Igl W, Lukas A, Bader A, Bailer S, Franke S et al (2010) Relationship between fear of falling and outcomes of an inpatient geriatric rehabilitation population–fear of the fear of falling. J Am Geriatr Soc 58:664–673. 10.1111/j.1532-5415.2010.02759.x 20345868 10.1111/j.1532-5415.2010.02759.x

[10] Montero-Odasso M, van der Velde N, Martin FC, Petrovic M, Tan MP, Ryg J et al (2022) World guidelines for falls prevention and management for older adults: a global initiative. Age Ageing 51:afac205. 10.1093/ageing/afac205 36178003 10.1093/ageing/afac205 PMC9523684

[11] McGarrigle L, Yang Y, Lasrado R, Gittins M, Todd C (2023) A systematic review and meta-analysis of the measurement properties of concerns-about-falling instruments in older people and people at increased risk of falls. Age Ageing 52:afad055. 10.1093/ageing/afad055 37211363 10.1093/ageing/afad055 PMC10200549

[12] Feng C, Adebero T, DePaul VG, Vafaei A, Norman KE, Auais M (2022) A Systematic review and meta-analysis of exercise interventions and use of exercise principles to reduce fear of falling in community-dwelling older adults. Phys Ther 102:pzab36. 10.1093/ptj/pzab236 10.1093/ptj/pzab236 34636923

[13] Chua CHM, Jiang Y, Lim DS, Wu VX, Wang W (2019) Effectiveness of cognitive behaviour therapy-based multicomponent interventions on fear of falling among community-dwelling older adults: a systematic review and meta-analysis. J Adv Nurs 75:3299–3315. 10.1111/jan.14150 31287182 10.1111/jan.14150

[14] Kruisbrink M, Crutzen R, Kempen GIJM, Delbaere K, Ambergen T, Cheung KL et al (2022) Disentangling interventions to reduce fear of falling in community-dwelling older people: a systematic review and meta-analysis of intervention components. Disabil Rehabil 44:6247–6257. 10.1080/09638288.2021.1969452 34511009 10.1080/09638288.2021.1969452

[15] Hu Y, Wang K, Gu J, Huang Z, Li M (2024) Effect of combined physical and cognitive intervention on fear of falling in older adults: a systematic review and meta-analysis. Arch Gerontol Geriatr 117:105173. 10.1016/j.archger.2023.105173 37713935 10.1016/j.archger.2023.105173

[16] Kumar A, Delbaere K, Zijlstra GAR, Carpenter H, Iliffe S, Masud T et al (2016) Exercise for reducing fear of falling in older people living in the community: Cochrane systematic review and meta-analysis. Age Ageing 45:345–352. 10.1093/ageing/afw036 27121683 10.1093/ageing/afw036

[17] Perry SW, Finch T, Deary V (2013) How should we manage fear of falling in older adults living in the community? BMJ Online 346:f2933. 10.1136/bmj.f2933 10.1136/bmj.f2933 23714190

[18] Meekes WMA, Leemrijse CJ, Korevaar JC, Stanmore EK, van de Goor LIAM (2022) Implementing falls prevention in primary care: barriers and facilitators. Clin Interv Aging 17:885–902. 10.2147/CIA.S354911 35686030 10.2147/CIA.S354911 PMC9171056

[19] Loganathan A, Ng CJ, Tan MP, Low WY (2015) Barriers faced by healthcare professionals when managing falls in older people in Kuala Lumpur, Malaysia: a qualitative study. BMJ Open 5:e008460. 10.1136/bmjopen-2015-008460 26546140 10.1136/bmjopen-2015-008460 PMC4636608

[20] Stevens JA (2013) The STEADI tool kit: a fall prevention resource for health care providers. IHS Prim Care Provid 39:162 26766893 PMC4707964

[21] McLennan C, Sherrington C, Tilden W, Jennings M, Richards B, Hill A-M et al (2024) Considerations across multiple stakeholder groups when implementing fall prevention programs in the acute hospital setting: a qualitative study. Age Ageing 53:afae208. 10.1093/ageing/afae208 39354814 10.1093/ageing/afae208 PMC11445322

[22] Fernandes TG, Silva KR, Guerra RO, Parente RCP, Borges GF, Freire Junior RC (2021) Influence of the Amazonian context on the frailty of older adults: a population-based study. Arch Gerontol Geriatr 93:104162. 10.1016/j.archger.2020.104162 32624196 10.1016/j.archger.2020.104162

[23] Esbrí-Víctor M, Huedo-Rodenas I, López-Utiel M, Navarro-López JL, Martínez-Reig M, Serra-Rexach JA et al (2017) Frailty and fear of falling: the FISTAC study. J Frailty Aging 6:136–140. 10.14283/jfa.2017.19 28721429 10.14283/jfa.2017.19

[24] Nicklen B, Delbaere K, Ellmers TJ. Is frailty associated with increased risk of concerns about falling and activity restriction in older adults? A systematic review. J Frailty Aging (**in press**)10.1016/j.tjfa.2024.10000239855888

[25] Cameron ID, Dyer SM, Panagoda CE, Murray GR, Hill KD, Cumming RG et al (2018) Interventions for preventing falls in older people in care facilities and hospitals. Cochrane Database Syst Rev. 10.1002/14651858.CD005465.pub4 30191554 10.1002/14651858.CD005465.pub4 PMC6148705

[26] Haines TP, Hill KD, Bennell KL, Osborne RH (2007) Additional exercise for older subacute hospital inpatients to prevent falls: benefits and barriers to implementation and evaluation. Clin Rehabil 21:742–753. 10.1177/0269215507079842 17846074 10.1177/0269215507079842

[27] Memon MA, Ting H, Cheah J-H, Thurasamy R, Chuah F, Cham TH (2020) Sample size for survey research: review and recommendations. J Appl Struct Equ Model 4:1–XX. 10.47263/JASEM.4(2)01

[28] Dillman DA (2011) Mail and internet surveys: the tailored design method—2007 update with new internet, visual, and mixed-mode guide. Wiley, Hoboken

[29] Thorpe C, Ryan B, McLean SL, Burt A, Stewart M, Brown JB et al (2009) How to obtain excellent response rates when surveying physicians. Fam Pract 26:65–68. 10.1093/fampra/cmn097 19074758 10.1093/fampra/cmn097

[30] Beatty PC, Willis GB (2007) Research synthesis: the practice of cognitive interviewing. Public Opin Q 71:287–311. 10.1093/poq/nfm006

[31] Willis GB, Anthony R, Artino J (2013) What do our respondents think we’re asking? using cognitive interviewing to improve medical education surveys. J Grad Med Educ 5:353. 10.4300/JGME-D-13-00154.1 24404294 10.4300/JGME-D-13-00154.1 PMC3771159

[32] Meadows K (2021) Cognitive interviewing methodologies. Clin Nurs Res 30:375–379. 10.1177/10547738211014099 33998325 10.1177/10547738211014099

[33] Belloni G, Bula C, Santos-Eggimann B, Henchoz Y, Seematter-Bagnoud L (2020) A single question as a screening tool to assess fear of falling in young-old community-dwelling persons. J Am Med Dir Assoc 21:1295-1301.e2. 10.1016/j.jamda.2020.01.101 32165062 10.1016/j.jamda.2020.01.101

[34] Denkinger MD, Igl W, Coll-Planas L, Nikolaus T, Bailer S, Bader A et al (2009) Practicality, validity and sensitivity to change of fear of falling self-report in hospitalised elderly—a comparison of four instruments. Age Ageing 38:108–112. 10.1093/ageing/afn233 19001557 10.1093/ageing/afn233

[35] Tennstedt S, Howland J, Lachman M, Peterson E, Kasten L, Jette A (1998) A randomized, controlled trial of a group intervention to reduce fear of falling and associated activity restriction in older adults. J Gerontol B Psychol Sci Soc Sci 53B:P384–P392. 10.1093/geronb/53B.6.P384 10.1093/geronb/53b.6.p384 9826971

[36] Wetherell JL, Johnson K, Chang D, Ward SR, Bower ES, Merz C et al (2016) Activity, balance, learning, and exposure (ABLE): a new intervention for fear of falling. Int J Geriatr Psychiatry 31:791–798. 10.1002/gps.4393 26729564 10.1002/gps.4393 PMC6339991

[37] Denneny D, Frijdal (nee Klapper) A, Bianchi-Berthouze N, Greenwood J, McLoughlin R, Petersen K et al (2020) The application of psychologically informed practice: observations of experienced physiotherapists working with people with chronic pain. Physiotherapy 106:163–73. 10.1016/j.physio.2019.01.014 10.1016/j.physio.2019.01.014 30930053

[38] Lenouvel E, Novak L, Biedermann A, Kressig RW, Klöppel S (2022) Preventive treatment options for fear of falling within the Swiss healthcare system. Z Gerontol Geriatr 55:597–602. 10.1007/s00391-021-01957-w 34590162 10.1007/s00391-021-01957-w PMC9587118

[39] Eckstrom E, Vincenzo JL, Casey CM, Gray S, Cosley K, Caulley J et al (2024) American Geriatrics Society response to the world falls guidelines. J Am Geriatr Soc 72:1669–1686. 10.1111/jgs.18734 38131656 10.1111/jgs.18734 PMC11187658

[40] Lenouvel E, Ullrich P, Siemens W, Dallmeier D, Denkinger M, Kienle G et al (2023) Cognitive behavioural therapy (CBT) with and without exercise to reduce fear of falling in older people living in the community. Cochrane Database Syst Rev 11:CD014666 37965937 10.1002/14651858.CD014666.pub2 PMC10646947

[41] Kruisbrink M, Delbaere K, Kempen GIJM, Crutzen R, Ambergen T, Cheung K-L et al (2021) Intervention characteristics associated with a reduction in fear of falling among community-dwelling older people: a systematic review and meta-analysis of randomized controlled trials. Gerontologist 61:e269–e282. 10.1093/geront/gnaa021 32267498 10.1093/geront/gnaa021 PMC8361503

