# Supplementary material for: Exploring how falls prevention practitioners assess and manage concerns about falling
Source: Eur Geriatr Med. 2024 Dec 17;16(1):219–27. doi: 10.1007/s41999-024-01127-2 (PMC11850553; doi:10.1007/s41999-024-01127-2)
Supplement: Supplementary file 1 — Supplementary file1 (DOCX 49 KB) [file 41999_2024_1127_MOESM1_ESM.docx]

Supplementary Materials

1. Checklist for Reporting Results of Internet E-Surveys (CHERRIES)

|  | **Checklist for Reporting Results of Internet E-Surveys (CHERRIES)** | |  |
| --- | --- | --- | --- |
| ***Item Category*** | ***Checklist Item*** | ***Explanation*** |  |
| **Design** |  |  |  |
|  | Describe survey design | Describe target population, sample frame. Is the sample a convenience sample? (In “open” surveys this is most likely.) | Page 3-5 |
| **IRB (Institutional Review Board) approval and informed consent process** |  |  |  |
|  | IRB approval | Mention whether the study has been approved by an IRB. | Page 3 |
|  | Informed consent | Describe the informed consent process. Where were the participants told the length of time of the survey, which data were stored and where and for how long, who the investigator was, and the purpose of the study? | Page 3 |
|  | Data protection | If any personal information was collected or stored, describe what mechanisms were used to protect unauthorized access. | Page 3 |
| **Development and pre- testing** |  |  |  |
|  | Development and testing | State how the survey was developed, including whether the usability and technical functionality of the electronic questionnaire had been tested before fielding the questionnaire. | Page 3-5 |
| **Recruitment process and description of the sample having access to the questionnaire** |  |  |  |
|  | Open survey versus closed survey | An “open survey” is a survey open for each visitor of a site, while a closed survey is only open to a sample which the investigator knows (password- protected survey). | Page 3 |
|  | Contact mode | Indicate whether or not the initial contact with the potential participants was made on the Internet. (Investigators may also send out questionnaires by mail and allow for Web-based data entry.) | Page 3 |
|  | Advertising the survey | How/where was the survey announced or advertised? Some examples are offline media (newspapers), or online (mailing lists – If yes, which ones?) or banner ads (Where were these banner ads posted and what did they look like?). It is important to know the wording of the announcement as it will heavily influence who chooses to participate. Ideally the survey announcement should be published as an appendix. | Page 3 |
| **Survey administration** |  |  |  |
|  | Web/E-mail | State the type of e-survey (eg, one posted on a Web site, or one sent out through e-mail). If it is an e-mail survey, were the responses entered manually into a database, or was there an automatic method for capturing responses? | Page 3 |
|  | Context | Describe the Web site (for mailing list/newsgroup) in which the survey was posted. What is the Web | Page 3 |

|  | **Checklist for Reporting Results of Internet E-Surveys (CHERRIES)** | |  |
| --- | --- | --- | --- |
| ***Item Category*** | ***Checklist Item*** | ***Explanation*** |  |
|  |  | site about, who is visiting it, what are visitors normally looking for? Discuss to what degree the content of the Web site could pre-select the sample or influence the results. For example, a survey about vaccination on a anti-immunization Web site will have different results from a Web survey conducted on a government Web site |  |
|  | Mandatory/voluntary | Was it a mandatory survey to be filled in by every visitor who wanted to enter the Web site, or was it a voluntary survey? | Page 3 |
|  | Incentives | Were any incentives offered (eg, monetary, prizes, or non-monetary incentives such as an offer to provide the survey results)? | Page 3 |
|  | Time/Date | In what timeframe were the data collected? | Page 5 |
|  | Randomization of items or questionnaires | To prevent biases items can be randomized or alternated. | N/A |
|  | Adaptive questioning | Use adaptive questioning (certain items, or only conditionally displayed based on responses to other items) to reduce number and complexity of the questions. | N/A |
|  | Number of Items | What was the number of questionnaire items per page? The number of items is an important factor for the completion rate. | Page 4 |
|  | Number of screens (pages) | Over how many pages was the questionnaire distributed? The number of items is an important factor for the completion rate. | N/A |
|  | Completeness check | It is technically possible to do consistency or completeness checks before the questionnaire is submitted. Was this done, and if “yes”, how (usually JAVAScript)? An alternative is to check for completeness after the questionnaire has been submitted (and highlight mandatory items). If this has been done, it should be reported. All items should provide a non-response option such as “not applicable” or “rather not say”, and selection of one response option should be enforced. | Page 11 |
|  | Review step | State whether respondents were able to review and change their answers (eg, through a Back button or a Review step which displays a summary of the responses and asks the respondents if they are correct). | Page 5 |
| **Response rates** |  |  |  |
|  | Unique site visitor | If you provide view rates or participation rates, you need to define how you determined a unique visitor. There are different techniques available, based on IP addresses or cookies or both. | N/A |
|  | View rate (Ratio of unique survey visitors/unique site visitors) | Requires counting unique visitors to the first page of the survey, divided by the number of unique site visitors (not page views!). It is not unusual to have view rates of less than 0.1 % if the survey is voluntary. | N/A |
|  | Participation rate (Ratio of unique visitors who agreed to | Count the unique number of people who filled in the first survey page (or agreed to participate, for example by checking a checkbox), divided by | N/A |

|  | **Checklist for Reporting Results of Internet E-Surveys (CHERRIES)** | |  |
| --- | --- | --- | --- |
| ***Item Category*** | ***Checklist Item*** | ***Explanation*** |  |
|  | participate/unique first survey page visitors) | visitors who visit the first page of the survey (or the informed consents page, if present). This can also be called “recruitment” rate. |  |
|  | Completion rate (Ratio of users who finished the survey/users who agreed to participate) | The number of people submitting the last questionnaire page, divided by the number of people who agreed to participate (or submitted the first survey page). This is only relevant if there is a separate “informed consent” page or if the survey goes over several pages. This is a measure for attrition. Note that “completion” can involve leaving questionnaire items blank. This is not a measure for how completely questionnaires were filled in. (If you need a measure for this, use the word “completeness rate”.) | N/A |
| **Preventing multiple entries from the same individual** |  |  |  |
|  | Cookies used | Indicate whether cookies were used to assign a unique user identifier to each client computer. If so, mention the page on which the cookie was set and read, and how long the cookie was valid. Were duplicate entries avoided by preventing users access to the survey twice; or were duplicate database entries having the same user ID eliminated before analysis? In the latter case, which entries were kept for analysis (eg, the first entry or the most recent)? | N/A |
|  | IP check | Indicate whether the IP address of the client computer was used to identify potential duplicate entries from the same user. If so, mention the period of time for which no two entries from the same IP address were allowed (eg, 24 hours). Were duplicate entries avoided by preventing users with the same IP address access to the survey twice; or were duplicate database entries having the same IP address within a given period of time eliminated before analysis? If the latter, which entries were kept for analysis (eg, the first entry or the most recent)? | N/A |
|  | Log file analysis | Indicate whether other techniques to analyze the log file for identification of multiple entries were used. If so, please describe. | N/A |
|  | Registration | In “closed” (non-open) surveys, users need to login first and it is easier to prevent duplicate entries from the same user. Describe how this was done. For example, was the survey never displayed a second time once the user had filled it in, or was the username stored together with the survey results and later eliminated? If the latter, which entries were kept for analysis (eg, the first entry or the most recent)? | N/A |
| **Analysis** |  |  |  |
|  | Handling of incomplete questionnaires | Were only completed questionnaires analyzed? Were questionnaires which terminated early (where, for example, users did not go through all questionnaire pages) also analyzed? | Page 4 |

|  | **Checklist for Reporting Results of Internet E-Surveys (CHERRIES)** | |  |
| --- | --- | --- | --- |
| ***Item Category*** | ***Checklist Item*** | ***Explanation*** |  |
|  | Questionnaires submitted with an atypical timestamp | Some investigators may measure the time people needed to fill in a questionnaire and exclude questionnaires that were submitted too soon.  Specify the timeframe that was used as a cut-off point, and describe how this point was determined. | N/A |
|  | Statistical correction | Indicate whether any methods such as weighting of items or propensity scores have been used to adjust for the non-representative sample; if so, please describe the methods. | N/A |

1. Recruitment materials

We are currently recruiting UK and Irish falls prevention healthcare professionals to complete an anonymous 10-15 min survey about assessing and managing fear of falling. This survey is on the topic of how to enhance the assessment and management of fear of falling in older people. To learn more information or to complete the survey, please follow: [INSERT SURVEY URL]

1. Questions used in the survey

**Question 1: Demographic information**

- In what country are you currently practising?
  - List: England, Wales, Scotland, Northern Ireland, Republic of Ireland, Other: please list

(Note: by practising in one of these 5 countries (UK + Ireland) will also be an inclusion criteria.)

- Age
- Gender (Drop down: Male, Female, I identify outside of the above options, prefer not to answer)
- In what type of service are you currently working (e.g., community setting, hospital acute services, Hospital long-term care services, Hospital outpatient/rehabilitation services, etc.)?
- What is your professional role?
- What service are you currently working in?
  - Falls service/clinic
  - Mental Health
  - Other: Please specify
- What is your current band (if appropriate)?
- How many years have you been qualified under your current role?
- In what country did you receive your qualification?
  - List: England, Wales, Scotland, Northern Ireland, Republic of Ireland, Other: please list
- What is the highest qualification that you have obtained?
  - Masters (Conversion)
  - Masters (Other)
  - Medical Doctor
  - NVQ
  - PhD
  - Undergraduate Degree
  - Other: Please specify

*This section will ask about formal and informal methods of assessing older adults’ concerns about falling in clinical practice.*

**Question 2:**

- How often do **personally** you see older adults within your current practice? (closed options)
  - Daily, Multiple times per week, Once a week, Once a month, Once every few months, Once a year, Every few years, I do not personally see older adults within my current practice

**Training**

**Question 3:**

- Have you received any formal training specifically to assist older people who are concerned/fearful about falling? (Y/N)
  - If yes, what training did you receive? Please list the type, number, and duration of training received (e.g., a single half-day workshop delivered by….)

**Question 4:**

- If yes, what training did you receive?
  - CPD
  - Degree module: Postgraduate
  - Degree module: Undergraduate
  - External Courses
  - In-service training
  - Webinars
  - Other: please specify

**Assessment of concern/fear about falling**

*This section will ask about formal and informal methods of assessing older adults’ concerns/fear about falling in clinical practice.*

**Question 5:**

- Do you currently use any **formal** tools (e.g., questionnaire) to assess concerns/fear about falling in older adults? (Y/N)
  - If yes…. what **formal assessment tools** do you use?
    - Short (7-item) Falls Efficacy Scale International (FES-I)
    - Full (16-item) Falls Efficacy Scale International (FES-I)
    - Icon FES-I
    - Modified Falls Efficacy Scale (MFES)
    - Activities-Specific Balance Confidence (ABC)
    - Survey of Activities and Fear of Falling in the Elderly (SAFFE)
    - Single-item assessment (please state the specific question asked and the possible answers that patients can provide)
    - Other (please state):_________________________________________
  - At what point(s) do you use these tools to assess concerns/fear about falling?
    - Intake/initial assessment
    - Monitoring of treatment (ie. during treatment)
    - Post-treatment assessment
  - Please describe how frequently you assess this on an ongoing basis
    - Every Month
    - Every two months
    - Every six months
    - Yearly
    - Other

**Question 6:**

- If no…. As you have indicated that you do not use formal assessments in your practice, please select from the list below for any potential reasons why this may be…
  - - Time constraints
    - Lack of knowledge/training about formal assessment tools
    - Lack of confidence using formal assessment tools
    - Not deemed relevant/important for my patients
    - I prefer to use informal modes of assessment: Please describe___________________________________________
    - Other: Please specify_________________________________

*This section will ask about any concerns or barriers surrounding current concerns about falling interventions*

**Concerns/Fear about Falling Interventions**

**Question 7:**

- Within your practice, how are concerns/fear about falling addressed? Please be as detailed as possible.
  - Formal psychological methods (e.g., standardised intervention such as CBT or educational programme): Please describe in as much detail as possible how concerns about falling are managed using this method (including the duration and frequency, e.g., 30 mins per week over 6 weeks).
  - Informal psychological methods (e.g., discussions with patient or their family): Please describe in as much detail as possible how concerns about falling are managed using this method (including the duration and frequency, e.g., 30 mins per week over 6 weeks).
  - Physical intervention (e.g. balance training etc) Please describe in as much detail as possible how concerns about falling are managed using this method (including the duration and frequency, e.g., 30 mins per week over 6 weeks).
  - We do not address concerns about falling in my practice. Please expand on what the barriers are that are preventing you from addressing CaF.

When considering the current interventions/methods used to address concerns/fear about falling in your practice, how would you rate the following…

**Question 8:**

Overall, how do the older adults engage with the intervention?

| Completely unengaged | Mostly Unengaged | Somewhat unengaged | Somewhat engaged | Mostly engaged | Completely engaged |
| --- | --- | --- | --- | --- | --- |
| 1 | 2 | 3 | 4 | 5 | 6 |

**Question 9:**

Please rate how effective you feel the intervention/s is at addressing concerns about falling?

| Extremely ineffective | Mostly ineffective | Somewhat ineffective | Somewhat effective | Mostly effective | Extremely effective |
| --- | --- | --- | --- | --- | --- |
| 1 | 2 | 3 | 4 | 5 | 6 |

- For those that select “somewhat-“, “mostly-“and “extremely- ineffective” are asked to list reasons why you feel the current interventions are ineffective

**Question 10:**

Overall, how acceptable is the intervention/s for the older adults to whom it is delivered?

| Extremely unacceptable | Mostly unacceptable | Somewhat unacceptable | Somewhat acceptable | Mostly acceptable | Extremely acceptable |
| --- | --- | --- | --- | --- | --- |
| 1 | 2 | 3 | 4 | 5 | 6 |

**Question 11:**

How confident do you personally feel in addressing concerns about falling?

| Not at all | Slightly unconfident | Somewhat unconfident | Somewhat confident | Fairly confident | Completely confident |
| --- | --- | --- | --- | --- | --- |
| 1 | 2 | 3 | 4 | 5 | 6 |

**Question 12:**

- What do you feel the main barriers are to clinically addressing concerns/ fear about falling?
  - - Difficulties in communication between departments
    - Difficulties in communication with the patient
    - Lack of confidence administering interventions
    - Lack of effective intervention options for my patients
    - Lack of knowledge/training about possible interventions
    - Lack of relevant interventions options for my patients
    - Time constraints
    - Other: Please specify_______________________________

*This section will ask about the treatment of concerns about falling in complex patients.*

**Complex Patient Needs**

**Question 13:**

- Do your patients with concerns/ fear about falling have other complex needs (e.g., frailty, sarcopenia, chronic pain, cognitive impairment, etc)?
  - - - None of the patients
      - Some of the patients
      - Most of the patients
      - All of the patients

**Question 14:**

- - - - - What are the top 3 most common complex needs seen in older adults who are concerned/fearful of falling?
  - Chronic pain
  - Cognitive impairment
  - Dizziness
  - Frailty
  - Mental Illhealth
  - Parkinson’s Disease
  - Sarcopenia
  - Stroke
  - Visual Impairment
  - Other

*This section will ask about the clinical responsibilities for concerns about falling.*

**Clinical Responsibilities for Concerns/Fear about Falling**

**Question 15:**

- - - - - Whose role do you think it is to **clinically assess** concerns about falling in older adults? (Please select all that apply)
  - Geriatrician
  - GP
  - Nurse
  - Occupational Therapist
  - Physiotherapist
  - Psychiatrist
  - Psychologist
  - Other

**Question 16:**

- - - - - Whose role do you think it is **to administer clinical interventions** to address concerns about falling in older adults? (Please select all that apply)
  - Geriatrician
  - GP
  - Nurse
  - Occupational Therapist
  - Physiotherapist
  - Psychiatrist
  - Psychologist
  - Other

**Question 17:**

- How effective do you feel the communication is between **different healthcare professionals** (e.g., psychologists, physiotherapists, occupational therapists etc.) when it comes to managing concerns about falling?

| Extremely ineffective | Mostly ineffective | Somewhat ineffective | Somewhat effective | Mostly effective | Extremely effective |
| --- | --- | --- | --- | --- | --- |
| 1 | 2 | 3 | 4 | 5 | 6 |

**Question 18:**

- - - - - If “somewhat-“, “Mostly-“ or “Extremely ineffective” is selected the following question appears: Please list the main barrier(s) for effective communication between professionals

**Question 19:**

- How effective do you feel the communication is between patient and healthcare professionals when it comes to managing concerns about falling?

| Extremely ineffective | Mostly ineffective | Somewhat ineffective | Somewhat effective | Mostly effective | Extremely effective |
| --- | --- | --- | --- | --- | --- |
| 1 | 2 | 3 | 4 | 5 | 6 |

**Question 20:**

**(Optional) Additional Comments Section**

Please feel free to share any additional commentary or insight on the subjects touched upon (or not) in the survey. As this is an exploratory study on experiences managing older adults’ concerns/fear about falling, any further commentary is greatly welcomed.

___________________________________________________________________________________________________________________________________________________________________________________________________________________________

1. Open-ended question answers for the different interventions used by healthcare professionals

| **Formal Psychological Methods** |
| --- |
| limited but on ward clinical psychology 1:1 session, several times overstay but not a standardised time frame |
| In past three months I have referred 3 patients out of 20 assessed for I talk cbt |
| Anxiety management |
| Education - in a 1/1 treatment if the client has spoken about COF or it is evident in their ax - then clients are reminded in their treatments about their current function and reasons for this concern. Sometimes education deals with it being a correct response and we refresh/practice the skills they need to do to allow them to feel confident and able to perform their task. Sometimes the education part is a refresher of methods in place to keep them safe and to give them independence in tasks and to reduce their COF. This could be weekly with set therapist, but HCA and SN will also be informed of tasks/methods/education tips that need daily support/reminding as needs apply. |
| Frailty clinic is an assessment service, so ppl with falls or fear of falling are given CSP falls information booklet with information referred to community physiotherapy for treatment (OTAGO and home hazard Ax) |
| referred to psychology |
| Workshops |
| SPIRIT/5 areas assessment where possible, integrated with physical interventions |
| **Informal psychological** |
| In A&E we only see patient once and would not be for long - 1-2hrs on average for whole assessment. We address falls verbally and give advice verbally to family and patient. E.G if family can provide supervision for a certain ADL that has prev resulted in a fall. Providing advice r.e alcohol intake (refer to alcohol services if needed), discuss fear of falling and whether this restricts engagement in ADL's etc. |
| alarms and systems to alert re falls are discussed and OT discussion with them re falls prevention and techniques to stand up from PT also re alarms and |
| During classes, there is an education element, where issues relating to health, and how that impacts balance or discussed. In addition, written material is given out to support the exercise booklet with additional exercises taught by later life training. |
| We discuss in clinic concerns regarding falling and try to address this, but time is a factor with clinics only 30 minutes for new patients and 15 for reviews |
| Information is gathered and then passed on to the therapy teams to enable them to rehabilitate patients at risk of falling and to provide equipment that may help minimise falls |
| brief discussion during the limited 15-20 mins for full assessment as a one off |
| Motivational speaking to patients and family, Reassurance, support as required |
| education and discussion with pt and family, adapt adls to boost confidence, equipment walking aids for confidence |
| Informal conversation and follow up on comments/concerns raised by patients and their families. No set method - depends on content of conversation |
| Discussed at each intervention session. This can range from a one-off sessions to several months of appointments to group exercise sessions. Advice always tailored specifically for each patient and adjusted according to their specific situation. |
| Discussion with patient and care givers re how to manage fear of falling, coping strategies once a fall happens, e.g., how to summon help, prevent further injury or long lie etc, up to 20-30 mins per therapy session |
| …if a client is showing COF with certain tasks and it's reducing their independence then the whole team (therapist, HCA, SN, activity coordinator) will be advised on current straggles to support the client with this. The client will be aware that team are all working together to support them so it could be daily and as needed. |
| Brief acknowledgement that fear is contributing to falling and need to continue to walk and engage in physical activity |
| Advice re how to prevent falls, handouts from csp- get up and go, falls prevention, reassuring re fears and ways to manage anxieties eg relaxation class |
| Discussion with patient. |
| Patients offered session with OT to discuss anxiety management strategies if appropriate. Usually only 1-2 sessions for 30-60 mins |
| 5 mins advise on fear of falling is a risk for falls itself. Pt encouraged to carry on 2ith activities on a safe way |
| Generally, reassurance given re care arrangements on discharge and onward referral to community therapy, discuss strategies for calling for help in event of fall - discharge with pendant alarms or falls sensors where appropriate, discussion of environmental safety measures such as lighting, footwear |
| As its physio sessions its incorporated rather than standalone 30min discussion. Separate talk at FaME falls group on fear of falling and strategies run by wider team (B4 exercise professionals)  educational sessions over 6 weeks |
| Active listening to patient reports, reflecting concerns for clarity, explaining how interventions will help to alleviate fears through improved performance, confidence, and abilities |
| Psychoeducation around concerns about falling, relaxation techniques, graded goal setting to overcome concerns, explain role of exercise in promoting confidence in mobility and reducing concerns about falling |
| Education sessions run by OT as part of MDT approach |
| Graded exposure, distraction techniques, education. |
| Workshops |
| At assessment and reassessment 60 mins each and additional sessions of concern is noted as a n issue |
| This is very dependent on the HCP. some will be very thorough, others very cursory |
| Informal discussions with patient and family. |
| graded exposure, risk enablement theory |
| **Physical intervention (e.g. balance training etc)** |
| Providing HEP, TUSS assessment, providing equipment such as a walking aid, referring to Home First for home falls risk assessments, referral to falls clinics. Do dizziness screens - L/S BP checks, medication checks etc. |
| after in patent therapy referred for falls assisted discharge team who continue inpatient home for 5 days post discharge |
| 60 mins group exercise per week over 38 weeks |
| I link every movement in class to a functional movement in their day-to-day life. I describe how it will help them and what specifically it links to. |
| Strength and balance training weekly with home exercise between sessions advised, in manageable chunks. Sessions are an hour long. |
| Balance training exercises for 36 weeks following Later Life PSI FaME programme. Backward chaining and exercises on the floor also covered in the programme. Plus 30 minute educational talks after every session on a falls related topic |
| Balance training 60 mins per week for 20 weeks face to face plus him exercise. Regular backward chaining included in the course. Specific functional training on areas participants are fearful of falling E.g. steps, bending and reaching, turning. |
| OTAGO exercises with client/class once a week with instructions for them to do twice more during week. About 20-30 minutes to complete.  Falls Management Exercises (FaME) for 1 hour, weekly over 25 weeks; Also advised to practice twice weekly; Home exercise balance training provided over 6 sessions, 30 - 40 minutes. |
| Balance training 15 min per week x 12 weeks |
| referral to PT |
| It depends on the patients individual responses to the FES. If Fear is linked to physical deficit then treatment could focus on Strength and balance, mobility etc. If Fear is linked to the environment, we could focus on environmental assessment and equipment provision. If fear is linked to anxiety, we can incorporate anxiety management/ pacing/ confidence building activities. If fear is linked to breathlessness we can explore Breathing techniques, fatigue management pacing etc. If fear is linked to social isolation we can link in with social prescribers, local activity and exercise groups etc. |
| Patients that are identified as having concerns about falling are given strengthening, range of motion exercises and balance exercises 30 minutes per week individually or are in most cases included in weekly exercises session led by the Physiotherapist. |
| 60 min PSI class, once a week for 12 weeks FaME programme. Brief informal education sessions delivered by PSIs at each class. No formal session on CaF |
| Physio for balance intervention currently at home. Group previously but has now been stopped. |
| Explain the purpose of exercises, advise that they are done with staff or competent family so that risk is reduced |
| See above. No fixed timescales, patients' needs are assessed on an individual basis and intervention provided accordingly. |
| Majority of therapy intervention in any given session will be based on physical rehab eg balance and gait re-ed, up to 45-60 mins weekly with each patient |
| OTAGO programme - 4 sessions of 3 mins once a week |
| BOS is usually a starting point for my sessions when someone has balance impairments. We discuss their current ability and what we are working to do with our exercise-based sessions. With a 1/1 example, the client would complete a focussed programme (our frequency is 2/wk ) and team would be aware of programme and provide additional sessions as appropriate. So, a HEP would be printed/laminated and left with client for team to follow during week and perform as per plan. As we visit weekly and ongoing - the session with therapist can be 15-20mins. Strength, dynamic and static balance programme are examples of components used. Objects with balloons, balls are introduced and balance progressions with hand supports. |
| Assessment in clinic then community therapies for 1 hrs weekly for 6 weeks |
| Physiotherapy strength and balance exercises, up to 45 mins daily for 6 weeks |
| We see patients intensively as outpatients usually 2-3 days a week for 2-3 hours of sessions. Set up with independent gym programme, tai chi classes, balance classes, obstacle course, hydro |
| Rehab facility - patients typically seen daily initially, working on mobility, strength, balance. May be given exercises to do independently/ with family support. Explain how exercise/ activity reduces risks of falling. Discuss normal for fall to impact confidence - lots of reassurance and encouragement to support regaining confidence. Education on other factors like postural hypotension and how to manage this, with more reassurance. Regular input until at mobility baseline - then seen about twice a week for maintenance, which may include mobility and or exercises. |
| Strength and balance exercises |
| Tailored to the client. Multiple balance training tools Hep strength training |
| Balance training - 30-40 minutes for 6- 8 weeks longer if required. Also do environmental checks, advice on footwear, providing walking aids, referral to dietetics if required. |
| Otago programme with rehab assistants over 6 months |
| No restrictions on input, it will be patient specific based on need. We offer a 12 week exercise group which is 1 hour per week, with encouragement to exercise in between and some general advice on falls. we encourage people to self manage with home exercise programme. |
| Pt ref for 6 - week falls prevention programme on the community. Previously falls classes were available, now 1:1 Otago training |
| Balance assessment and training. No consistent duration and frequency as it is dependent on the ward caseload. Balance training usually done when medically fit for discharge and waiting care on discharge. Onward referrals completed. |
| Strength and gait practice while on ward daily. Advice on correct use of walking aids, safe transfer methods, waiting for AO1 where deemed necessary, use of tech to get help in event of fall (mobile, pendant alarm, fall sensors) |
| Balance programme to challenge and incorporate specific multifactorial issues ie vestibular hypofunction. Weekly sessions at home around 1 hour can be alternated with phone appts with focus on behaviour change and incorporating activity/ balance ex in daily plans. FaME group 90min weekly for 24 weeks and incorporates social time - peer support been really helpful re fear of falling. |
| sessions over 6 weeks with on going plan |
| As I am a vestibular specialist we perform all canal repositioning manoeuvres to address BPPV, standing balance training, gaze stabilisation exercises, strengthening exercises when needed, medication management (in conjunction with GP/Consultant) e.g. SSRI use for Persistent Postural Perceptual Dizziness or deprescribing to improve postural hypotension. These are individually tailored so if the problem is BPPV it could all be done in one 30 minute session but if medically complicated, it could be done at each session for up to 6 visits. |
| concerns about falling are discussed as part of OTAFO home exercise programme (6 home visits and 6 phone calls). Also deliver 17 week FaME/PSI programme which appears to improve concerns about falling but this needs to be specifically assessed. |
| Standard postural stability exercises plus the use of the Biodex balance platform. Graded exposure and practice of anxiety-provoking situations. Patient-centred discussions on the way forward. |
| Strength and balance programme based on OTAGO |
| Refer to falls clinic and strength and balance classes. - strength and balance are for 6 weeks once a week class |
| One session a week with an additional home exercise plan. Tailored and progressed balance interventions based on formal assessment using Berg balance scale. Attend usually for around 8 sessions or 12 if go to our class. |
| Provision of HEP programmes for balance, completed independently at home by individuals with review as necessary at the clinic |
| Balance training in situations where patient is most concerned/fearful. Ideally attend for 1hour per week for 6 weeks |
| Daily physiotherapy gym rehab sessions while an inpatient. 20-30 mins |
| Balance physio (outpatients), typically seen every 2 weeks for 8-12 sessions |
| Home exercise programme and group sessions |
| Weekly balance class 60 mins plus diary to do exercises 2x week at home |
| 1 hr in the community, plus homework |
| 12 week home exercise program based on OTAGO. |
| aids, exercises, balance training |
| **We do not address concerns about falling in my practice.** |
| Prior to the COVID pandemic there was a multi-disciplinary team providing a falls clinic, post COVID there have been staffing challenges and this clinic has not reset. There is a strength and balance programme delivered by the healthy living centres, fear of falling is currently not assessed as an outcome measure due to staffing resources and time required to administer and follow up on fear of falling within existing resource allocation. |
| …we also refer externally to Healthy Minds who address CaF with talking therapy In my experience this does happen too. As our services may not allow time to focus on this |
| assessment service only. Advise and booklet given and Pt is referred fo4 treatment to community physio |
| My service is often a short term reablement support service and often works to keep patients safe with formal falls assessment happening if more than 2 falls in 6 months. The falls team is however spread very thin in coverage and cannot actually offer rehab but short safety assessments and interventions |
